# Cystic Tumor of the Lower Jaw, with Giant-Celled Elements

**Published:** 1870-12

**Authors:** 


### ARTICLE XI.

### *Cystic Tumor of the Lower Jaw, with Giant-celled Elements.*

James H. B., 14 years of age, of Chester, Pa., came to the clinic on the 12th of October, on account of enlargement of the lower jaw of from two to three years' duration. His chin was a little prominent, especially on the left side; and on opening his mouth, the bone was seen to be expanded, thereby forming a tumor of the ovoïdal figure and of regular outline, extending from the canine tooth of the right side to the first molar of the left. The alveolar border of the growth was soft, and crackled like parchment when indented. The gum was natural in color; he had lost several teeth over the tumor, and the remaining ones were loose. No cause could be assigned for its appearance; it was devoid of pain except a slight aching during the past three weeks; its progress had been slow, but within the past three months it had about donbled itself. His grand-aunt died of cancer of the breast.

Having diagnosed a cystic tumor, Professor Gross made an incision into it on its anterior surface with an ordinary scalpel, and a thin brownish fluid escaped. There was a large mass of substance resembling fungous granulations attached to the inner walls of the cyst, which was scraped away with the chisel. The operation was attended with profuse hemorrhage; but it was promptly controlled by plugging the cavity with cotton wet with Monsel's solution.

The central portion of the mass bore a suspicious resemblance to encephaloid, and, on microscopic examination by Dr. W. W. Keen, he found "the small scraps of the central portion of the tumor, which were gouged out, looked exactly like the heart's muscles, and were scarcely less dense. When teased out, 'myeloplaxes,' or 'giant-cells,' or 'multi-nucleated' cells, were seen. The giant-cells formed, probably the fourth r fifth of the entire mass, and were very remarkable for heir bizarre forms and numerous prolongations or processes.

The number of nuclei was from ten to thirty, and nearly all possessed one, and often two, brilliant nucleoli.

"The spindle-shaped and other forms of caudate corpuscles, and round or ovoid cells, constituted the remainder of the mass. These, also, were generally nucleated and nucleolated. Scarcely any intercellular tissue was observed, and fatty granules were very rarely found to any extent, save in two or three places. So far as could be judged without injection, the mass was not very vascular."

The plug was allowed to remain for several days, until it had become loosened by suppuration, when it was removed, and a piece of patent lint wet with sweet oil was substituted. After a few days, the tents were dispensed with, and the cavity was then syringed out every few hours with a weak solution of permanganate of potassa. There was a little erysipelas after the operation, with difficulty in swallowing and a circumscribed hardness beneath the chin; but it disappeared under the usual treatment, and he was discharged, feeling quite comfortable, on October 26.

He reported at the clinic, on November 5, in good general health, and with the cavity in the jaw much diminished in size.—*Clinic of Prof. Gross. Med. Times.*

---

